# Long-Term Survival after Surgical Resection and Radiotherapy for Anterior Mediastinal Neuroblastoma in an Older Patient: A Case Report

**DOI:** 10.70352/scrj.cr.25-0540

**Published:** 2025-11-26

**Authors:** Ayako Hirai, Kiryoku Kanekatsu, Tomoya Kato

**Affiliations:** 1 Department of Thoracic Surgery, Fukushima Rosai Hospital, Iwaki, Fukushima, Japan; 2 Department of Pathology, Fukushima Rosai Hospital, Iwaki, Fukushima, Japan

**Keywords:** neuroblastoma, adult, anterior mediastinum, surgery, radiotherapy

## Abstract

**INTRODUCTION:**

Neuroblastoma is commonly seen in children younger than 5 years but is extremely rare in adults. There are only 23 reported cases of mediastinal neuroblastoma in adults, and no standard treatment strategy has been established.

**CASE PRESENTATION:**

A 79-year-old man was referred to our hospital for the investigation of an abnormal shadow observed on a routine chest radiograph. CT revealed a 5.5 × 5.0-cm mass in the anterior mediastinum, and fluorodeoxyglucose-PET demonstrated increased fluorodeoxyglucose uptake. Surgical resection was performed to obtain a definitive diagnosis and local control. Histopathological examination confirmed that the mass was a poorly differentiated neuroblastoma with invasion into the surrounding mediastinal fat. Postoperative radiotherapy was administered. The patient remains alive without recurrence at more than 5 years after surgery.

**CONCLUSIONS:**

Neuroblastoma arising in the anterior mediastinum of adults is extremely rare and the long-term prognosis remains unclear. Complete resection followed by radiotherapy may contribute to prolonged disease-free survival in selected adult patients.

## INTRODUCTION

A neuroblastoma is an embryonal tumor derived from the neural crest cells of the sympathetic nervous system that typically affects children younger than 5 years. However, neuroblastoma is extremely rare in adults, with only 23 reported cases of mediastinal neuroblastoma in adults to date.^1–20)^ While treatment protocols for pediatric neuroblastoma are well established and tailored according to risk classification, there is no consensus regarding the optimal treatment in adults. Some reports state that long-term survival of adults with neuroblastoma is achieved through a combination of surgery and adjuvant therapies.^3,18)^ We report the case of a 79-year-old man with an anterior mediastinal neuroblastoma who underwent complete surgical resection followed by radiotherapy and has remained recurrence-free for more than 5 years.

## CASE PRESENTATION

**Fig. 1:**
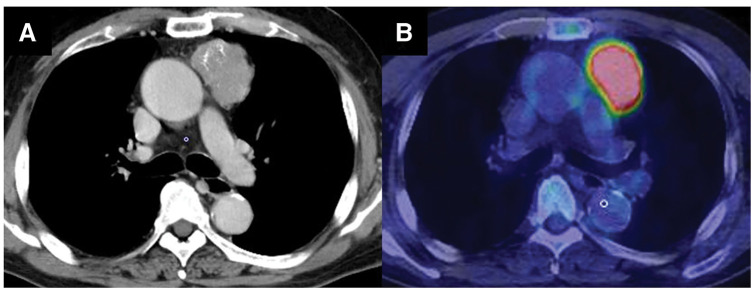
Preoperative images of the tumor. (**A**) Chest CT reveals a 5.1 × 5.0-cm well-defined mass in the left anterior mediastinum with calcification. (**B**) Fluorodeoxyglucose-PET shows increased fluorodeoxyglucose uptake with a maximum standardized uptake value of 12.0.

A 79-year-old man was referred to our hospital after an abnormal shadow was detected on a routine chest radiograph. Chest CT revealed a 5.1 × 5.0-cm well-defined mass in the left anterior mediastinum with internal calcification (**Fig. 1A**). Fluorodeoxyglucose-PET showed increased fluorodeoxyglucose uptake with a maximum standardized uptake value of 12.0 (**Fig. 1B**). The serum concentrations of the tumor markers α-fetoprotein, β-human chorionic gonadotropin, soluble interleukin-2 receptor, neuron-specific enolase, squamous cell carcinoma antigen, and cytokeratin 19 fragment were within normal ranges.

Given the absence of metastatic findings, we suspected a thymic tumor, such as thymoma or thymic carcinoma, and proceeded with surgical resection. The patient underwent total thymectomy with tumor removal via a median sternotomy, considering the possibility of combined pericardial resection. Intraoperatively, there was mild adhesion to the pericardium but no gross invasion, and the pericardium was preserved. A frozen section examination suggested type AB thymoma, as the tumor consisted of a type B-like lymphocyte-rich area and a type A-like lymphocyte-poor area. These components formed discrete lobules that were partly intermingled. Based on the diagnosis of thymoma, we determined that total thymectomy with removal of the tumor and surrounding mediastinal fat was sufficient, and pericardial resection was not required. The postoperative course was uneventful, and the patient was discharged without complications.

**Fig. 2:**
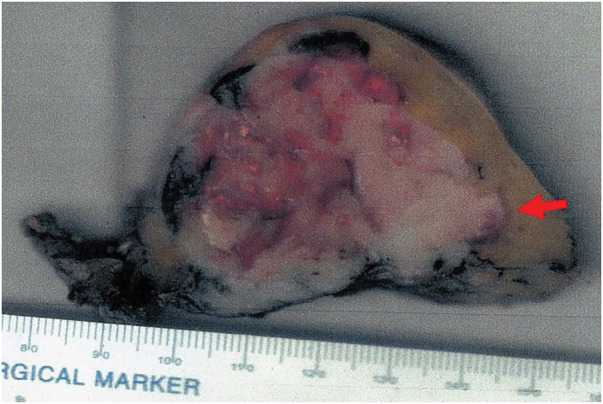
Photograph of the resected tumor. The tumor is soft, white, nodular, mostly encapsulated, and measures 5.5 × 5.2 cm. There is focal extension into the surrounding mediastinal fat (red arrow).

**Fig. 3:**
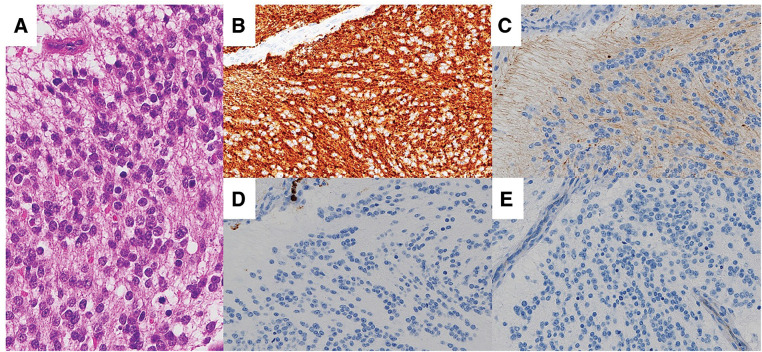
Histologic and immunohistochemical analyses. (**A**) Microscopically, the tumor consists of small round cells with hyperchromatic nuclei. The tumor is positive for (**B**) synaptophysin, and negative for (**C**) cytokeratin AE/AE3, (**D**) CD45, and (**E**) CD99.

The resected mass was a soft, white, nodular tumor that measured 5.5 × 5.2 cm (**Fig. 2**). The tumor was mostly encapsulated but showed focal extension into the surrounding mediastinal fat. Microscopically, the tumor consisted of small round cells with hyperchromatic nuclei arranged in lobular patterns, and was surrounded by a thin fibrous capsule (**Fig. 3A**). Residual thymic tissue was present around the tumor. The tumor had infiltrated into the surrounding mediastinal fat tissue, but negative surgical margins were achieved.

Immunohistochemistry revealed positivity for the neuroendocrine markers synaptophysin (**Fig. 3B**), chromogranin A, CD56, and neuron-specific enolase. The tumor cells were negative for epithelial markers (cytokeratin AE1/AE3 and EMA), lymphoid markers (CD45), and markers of primitive neuroectodermal tumors (CD99) (**Fig. 3C**–**3E**). Based on these findings, the final diagnosis was a poorly differentiated neuroblastoma.

We determined that complete resection was achieved without the pericardial resection, although the microscopic tumor margin on the pericardial side was close. With a diagnosis of neuroblastoma, the tumor was malignant and had infiltrated into the surrounding mediastinal fat tissue, leading us to consider the possibility of occult residual tumor on the pericardium. On a joint conference with a radiologist and a pathologist, it was concluded that the tumor originated in the anterior mediastinum and showed histologic continuity with the residual thymic tissue. On the basis of thymic carcinoma treatment guidelines that weakly recommend adjuvant radiotherapy for Masaoka stage II tumors,^21)^ we administered adjuvant radiotherapy (50 Gy in 25 fractions). Radiation pneumonitis was detected within the irradiation field; however, the patient did not develop any significant clinical symptoms. The patient has remained alive and disease-free for more than 5 years since treatment.

## DISCUSSION

Neuroblastoma is an embryonal tumor arising from the neural crest cells of the sympathetic nervous system and is primarily a pediatric malignancy that rarely occurs in adults. According to a SEER (Surveillance, Epidemiology, and End Results) database analysis (1973–2010), only 216 of 3818 neuroblastoma cases involved adults (age ≥18 years), and 35 of 3818 neuroblastoma cases involved older patients (>60 years).^12)^ Among these, only 2 of 3818 patients (0.05%) with a mediastinal tumor were older than 60 years.^12)^ Furthermore, the 5-year disease-specific survival rate of older patients with neuroblastoma was 40%.^12)^

Neuroblastomas commonly arise in the adrenal gland, but can also develop in the neck, extremities, and thoracic cavity. The International Neuroblastoma Pathology Classification categorizes neuroblastic tumors into 3 main subtypes: neuroblastoma, ganglioneuroblastoma, and ganglioneuroma.^22)^ Histologically, neuroblastomas are composed of small round blue cells, and immunohistochemical analysis is required to differentiate neuroblastomas from other small round cell tumors, such as thymoma, malignant lymphoma, and primitive neuroectodermal tumor. The key markers used to differentiate neuroblastomas from other round cell tumors include cytokeratins (for thymic tumors), CD45 (for lymphoid malignancies), and CD99 (for primitive neuroectodermal tumor).^18)^

The treatment strategy for pediatric neuroblastoma is risk-adapted, with options including observation, surgery, chemotherapy, radiotherapy, or multimodal approaches based on age, stage, histology, and tumor biology.^23)^ However, owing to the scarcity of adult cases, there is currently no standardized treatment regimen for adult neuroblastoma. Rogowitz et al. reported that intensive pediatric protocols are poorly tolerated by older patients. The discontinuation of these protocols has most likely contributed to the improved survival trend observed among older patients with neuroblastoma over the past decade.^12)^ Therefore, surgical resection remains the mainstay of treatment.

In previous studies, 21 of 23 adults with mediastinal neuroblastoma underwent surgical resection.^1–20)^ Four of these adult patients received perioperative therapy: one received neoadjuvant chemotherapy^16)^ and three received radiotherapy.^3,5,18)^ Among these, only 1 of 23 patients was reported to have a long survival without relapse,^15)^ while three were reported to have disease recurrence.^6,9,14)^ Almost all patients were followed up for only 3–24 months; however, late recurrence at 4 years after surgery was reported in one case,^14)^ suggesting that long-term follow-up is necessary.

Three of 21 patients who underwent resection received adjuvant radiotherapy. Among them, two patients had tumors measuring 10 and 7 cm, respectively, and achieved R0 resection, while one patient had a 3.6 cm tumor but underwent R1 resection. They remained disease-free at 18, 18, and 13 months of follow-up, respectively.^3,5,18)^ Among the three patients developed had disease recurrence, one patient died 12 months after surgery due to local and distant metastases, another developed local and pulmonary metastases 7 months after surgery, and the remaining patient developed a local recurrence 4 years after surgery. The tumor size of the first patient was not specified, whereas that of the second and third patients was 5 and 4.7 cm, respectively.^6,9,14)^ All patients who experienced recurrence had not received adjuvant therapy and developed local recurrence. In contrast, the patients who received adjuvant radiotherapy remained recurrence-free for at least 13–18 months of follow-up. The tumor size in these patients was not larger than that in patients without recurrence. According to limited available literature and expert opinion, surgical tumor resection should be followed by adjuvant radiotherapy and/or chemotherapy, depending on the margin status and the extent of disease.^18)^ In our case, as the microscopic tumor margin was close, we considered the possibility of occult residual tumor on the pericardium. To achieve complete local control, adjuvant radiotherapy was deemed necessary.

For older patients, particularly those aged around 80 years or older, the invasiveness of therapy may sometimes worsen the prognosis. Surgical resection is the mainstay of treatment for neuroblastoma, and negative surgical margins and the absence of lymph node involvement can result in long-term survival. Chemotherapy regimens designed for pediatric patients are often too intensive to be tolerated by older patients. Neuroblastoma is a malignant tumor with a poor prognosis, and in our case, we considered the possibility of occult residual tumor. We believe that long-term survival was achieved by combining surgery with adjuvant radiotherapy in a 79-year-old patient without complications. Our experience suggests that surgery followed by adjuvant radiotherapy may be an effective treatment strategy that contributes to favorable long-term outcomes in selected adult patients with neuroblastoma. Our patient is now 84 years old and remains alive without recurrence more than 5 years after surgery.

## CONCLUSIONS

A neuroblastoma arising in the anterior mediastinum is extremely rare in adults, and the long-term clinical behavior of such tumors remains uncertain. Our experience suggests that surgery followed by adjuvant radiotherapy may be an effective treatment strategy that contributes to favorable long-term outcomes in selected adult patients with neuroblastoma.

## DECLARATIONS

### Availability of data and materials

The dataset supporting the conclusions of this article is included within the article.
